# Mechanics in Dentistry

**Published:** 1892-12

**Authors:** W. G. A. Bonwill

**Affiliations:** Philadelphia


### MECHANICS IN DENTISTRY.—IS DENTISTRY A SCIENCE OR A PROFESSION?[1]

BY W. G. A. BONWILL, D.D.S., PHILADELPHIA.

A VERY unpalatable subject is this I have chosen,—"Mechanics in Dentistry." There is no other word in dental language that is held, to-day, in such disrepute. Every year there is fresh venom hurled against it, or any reference thereto, and it would seem to have fallen from the high eminence which it had in the early days. The good old name has a very strange synonyme,—prosthesis,—which might be well if it were confined to the supply alone of artificial dentures. A moment's reflection, however, will show that when we attempt to divide dentistry into operative and prosthetic or mechanical, we do injustice to our profession, and we assume a false position for our great art, which is founded almost exclusively in our ability as mechanicians, and without which, to a great degree, we would be helpless.

---

[1] Read before the Odontological Society of Pennsylvania, Saturday, October 8, 1892.

It has become such a disgrace to be known as a mechanic that it is amusing to see, not only dentists, but nearly every man upon the street, or wherever you may go, taking to the English mode of carrying the arms bent at a decided angle at the elbows, and the thumb projecting from the wide-open hand at as great a tangent as possible. You may be curious to know what this means. Well, I am responsible for the version and why originated.

The very act of using the hands to grasp the handle of a hammer, or lift a weight, necessitates the closing of all the fingers and the thumb over them, while the arm is usually, when lifting, nearly in a straight line. These finally give to the individual, when walking, arms that assume a line parallel and vertical with their body, with the hands mostly closed. See the application. A gentleman found with his arms bent at the elbows and thrown as far as possible—gracefully—from contact with the body, his hands with all fingers outstretched and thumb at a right angle with the index finger, will be known as one who never grasped an instrument or extended his arms straight to lift any object. The poor mechanic will be found invariably to take on exactly the opposite. He has never heard of "Hogarth's line of beauty." You may smile at this, but there is too much truth in it. I do not object to graceful positions, but teach the mechanic what it means and let him be an artist.

Students generally feel this tendency of our profession, and it is almost impossible to get them to prosecute this branch of dentistry. Unless they are compelled to learn laboratory work first and thoroughly, they never become efficient in the mechanical department. Once permit them to go to a dental college, where they are allowed the first session to handle delicate instruments over the operating-chair instead of rough tools, the hammer and the "anvil chorus" is no longer heard.

Before we touch upon science, let us ask whether we are a profession? What is its meaning?

The three learned callings are theology, law, and medicine.

The occupation, if not mechanical or agricultural, is placed as a profession. If you can make this of dentistry I am unable to interpret the meaning of our lexicons. The teaching of mathematics does not make it a profession any more than teaching mechanics. We cannot, then, arbitrarily place ourselves before the world as a profession, as now understood; but we are artists, artisans; or we can, more properly, be called scientific artisans.

What is science?

Science is a systematic and orderly arrangement of knowledge.

Facts and truths are the ultimate principles.

Pure science is a knowledge of forms, causes, or laws.

"While both science and art are synonymous, as they investigate truth, science is restricted to the inquiry for knowledge and art for the sake of production."

"The most perfect state of science will be the high and accurate inquiry, and the perfection of art the most apt and efficient system of rules,—art always throwing itself into the form of rules."

What are the sciences which have been so long recognized as seven in number?

Grammar, logic, rhetoric, arithmetic, geometry, astronomy, and music.

You perceive that medicine, law, and theology are not mentioned. They are professions.

You do not find mechanics among the seven. Yet mechanics is made up of geometry and arithmetic, placed together and made efficient and demonstrable by that higher power—God-like in itself—that conceives, designs, and creates, and which is of more importance than any and all of the others.

Boyle says that mechanics, in a larger sense, is for those disciplines that consist of the application of the pure mathematics to produce or modify motion in inferior bodies.

Huxley says, in the *Nineteenth Century*, "Newton defined the laws, rules, or observed order of the phenomena of motion which come under our daily observation with greater precision than had been before attained, and by following out with marvellous power and subtlety the mathematical consequences of these rules he almost created the modern science of pure mechanics."

Newton, in the preface to his "Principia," says, "The ancients made great account of the science of mechanics in the investigation of natural things, and considered mechanics in a twofold respect,—as rational, which proceeds accurately by demonstration, and practical.

"To practical mechanics all the manual arts belong, from which it took its name.

"To describe right lines and circles are problems, but not geometrical ones. The solution of these problems is required of mechanics, and by geometry the use of them, when solved, is shown. Therefore geometry is founded on mechanical practice, and is nothing but that part of universal mechanics which accurately proposes and demonstrates the art of measuring. Rational mechanics will be the science of motions."

He finally says, "I wish we could derive the rest of the phenomena of nature by the same kind of reasoning from mechanical principles."

Thus you see this great master's high estimation of mechanics, and he clearly shows that geometry and mathematics are the two simples that, when combined, give a result that has builded worlds, and without which Newton could not have discovered gravitation, nor could Kepler have formulated his "three laws of motion" which regulate the universe of suns and stars.

Tyndall said of Goethe, in scientific pursuits, that he was so devoid of mechanical principles and the minute details of the powers of construction that he was an ignis-fatuus, and unworthy to be followed.

What gave to the Egyptians the glory that to this day crowns their everlasting works in architecture and their tombs? Mechanics. Their monuments have never been excelled in perfect symmetry and beauty, and their pyramids show no lack of comparison in their calculations in the distance of the sun from the earth with the astronomers of to-day.

Where would they have been in history but for their mechanical productions, that promise to continue for thousands of years? What does the history of all nations have to show of greater interest and more lasting in its results than the mechanism displayed by its peoples? What could Napoleon have said when in Egpyt with his army at the foot of the great pyramids that would have created more enthusiasm than when he most poetically exclaimed, "Forty centuries look down upon you!" What if he had said the work of one hundred thousand poor mechanics, hod-carriers, stonemasons, carpenters, blacksmiths, and artists look down upon you? Go ask the architects of all those wonderful temples on the Nile how they moved those immense granite blocks from the quarry, and how raise them until pile on pile had massed a structure, the mode of doing which the wise mechanics of to-day cannot explain?

Ask the Roman Cæsar what gave him his power in battle? How could those magnificent pieces of masonry have been accomplished but for the skilled mechanics? Coming down to our own times, where is Watt? He lies entombed in Westminster Abbey, by the side of all that is honored in literature, art, science, and war. He was a mechanic!

Sir Christopher Wren. Is his name not upon every lip? What could he have done as a great architect without his inspired mechanical ability?

What has the printing-press done for mankind? It required a mechanic not only to make the type but the press. Is there any monument to the maker of these? Yes. Where is the monument to the dentist?

Morse needs no monument to tell those who may come four thousand years hence of the apparatus invented by him.

Ask Franklin, that universal genius, whether he would wish us to call him a mechanician?

We can ask the *Freemason*. His craft has come down to us through the course of time. No record shows its beginning. We see it to-day in man's memory only as it was in the inception. No tablet has written upon it a word to show its secrets, and yet they are emblazond on everything from the bottom to the highest pinnacle of the Mason's work. "Geometry is Masonry upon a general scale." Who does not consider it a high honor—the highest—to be admitted into its hallowed courts? Who of you that have officiated in its halls and chambers but is impressed at every "step" and every "halt" of the sacredness and importance of the science taught? Labor is engraven upon every panel of the doors that stand guard at "Who comes here?"

Ah, my fellow-members of the dental craft, how much longer will you persist in your ignorance and stupidity? I cannot lose sight of the days in my youth when I once made the jack-plane curl up the shavings to my delight, and how I turned these hands to account in the carpenter-shop, or the cabinet-makers' department, and when I could help to build a house, make a cradle or a coffin, chisel out a gun-stock, or make a blacksmith's or a church-organ bellows; or when there was nothing of this for me to do, I could go into a country store and measure the molasses or the calico. I am glad now of that varied experience.

I am very proud of mechanics when I look around and see men in dentistry who were once poor carpenters, and others engaged in other forms of mechanical trades, attempting to relegate mechanical dentistry to the shades. I feel sure that they never became masters of the art.

For all that is "true" in mechanical dentistry, what, on the other side, is false? We hear of little else at the present time but "science." They attempt to put down every man who has anything to say upon any other subject, and remind him that "if we would elevate our profession" we must take up the subjects of Remedies and Bacteriology. We must no longer fill teeth and resort to the mechanical manipulators.

Professor Miller, of Berlin, in an article read before the Southern Dental Association in 1890, or 1891, spoke of Arthur and Bonwill, who were the advocates of mechanical skill in dentistry, and asserted that it was not scientific to do as they were doing; that the mechanic had no place with the scientific men.

A better example cannot be given than the action of the four honorary American presidents of the International Medical Congress in Berlin, in 1890, Dental Section. The Committee on Essays invited me to read a paper on the Geometrical and Mechanical Laws of Articulation, with the understanding that I was to be there in person. I wrote to two of the honorable body in regard to it, and was advised by them to decline and not be present at the Congress; that I had sufficient honor in having received an invitation. I asked the reasons for such advice. Their argument was that Bonwill was not a scientific man, nothing but a mechanician and inventor, and was working his brain for all he could get out of the profession. A third one, who was interviewed by another party for me, said that they did not wish to have any dentist who could not talk pure science; that at the International Congress in 1880 in London, and at Washington in 1886, the foreign dentists had proven too much for the Americans, and demonstrated their inferior ability in practical scientific work, and that no man would be invited before the Berlin Congress unless he was a good scientific man.

Sir Astley Cooper said that medicine is a science founded on conjecture and improved by murder.

And so bacteriology in dentistry has not yet saved the first human tooth from the ravages of caries, nor has it in the future any bright ray of hope that it will accomplish all that the scientific dentist would have us believe.

If working our brains over such hypothetical problems will make us scientific dentists, while we are permitting the teeth to go to destruction through our present practice, let us hail it; but, at the same time, we would do well not to ignore that true branch of science—mechanics—which has from the inception been the only fixed and certain basis of attacking and battling with caries. We cannot practise the science of dentistry when we attempt to throw overboard its sheet-anchor,—*manual labor.*

So long as we cannot save human teeth by the theory of "germ treatment," but are compelled to labor with our hands, we are doomed to be looked upon as craftsmen and no better than the trades.

The mere experimenter, without any definite results in any line of work, is not a scientific man. Science is absolute and unchangeable. It is truth! It is an ultimate principle! There is but one result.

I would not have you understand that all experimenting in the secret chambers of nature should be denied. But what I do protest against is, that when scientific men have failed to save human teeth by certain known and acknowledged treatments and practices, they should assume to be the umpires to decide whether the present system, founded almost entirely upon mechanism, should be set aside.

When the teeth become so worn, where germs have had no part in the destruction, what can we look to as our alternative? Bacteriology?

The mechanical dentist is a fixture in our profession, and it would be well to make yourselves competent to be experts in your art, that you can stand in the eyes of the best of society on equal footing with the jeweller, diamond-setter, and cutter. The electrician could not do his work without the steam-engine to drive his dynamo.

The field of mechanical dentistry to-day is unique; and, while we have no system that has been universally adopted, we have done enough to place us as far on the record of progressive dentistry as has the so-called operative department.

While I am no friend to bridge-work as practised, yet it has taught a few men what we so much stand in need of,—manipulative skill.

Crowning has been one of the greatest steps in dentistry so far as pure artificial and mechanical work is concerned; but even this you will find has been most wofully abused by many of our craft, in cutting off the crowns of natural teeth that could have been saved with amalgam for years.

The truly competent dentist who is a born mechanician will seldom have to resort to a crown of any kind. The more we look at the subject will we find that dentistry is a medley, and no man can be competent to practise it who has not been educated in all its departments to do one as well as another.

What is it that will place us among the world's exemplars, and give to us, when dead, a place worthy to be remembered? One man from our ranks has been honored by a public monument, the discoverer of anæsthesia; and if it had been raised while he lived, I am not sure but he would have been abused that he did not give at once his discovery.

The first step to recognition by the public will be when we have, as I have intimated, sought truth for its own sake. Selfishness, envy, jealousy, and ingratitude can have no place in a body of men who labor for the general good. Let us be fair and, when one of our craft has given something original, and it proves a marked advance, place his name, while he lives, on the record. It will be in time a milestone in the records of dental history.

The next most important step is for the live men of our art to visit the great centres of trade and where machinery is developed, and watch the matchless system by which the minutest part is made, and also made interchangeable. Go into the draughtsman's department and scan each line and curve on that chart, where lies in perfect symmetry every portion of the huge engine to be born of that engineer's skill. Go and see it put together, and note whether every bolt and pinion does not fit with absolutely mathematical precision. System, order, conception, design, creation,—without these all would be chaos. Without the laws of geometry and mathematics, with a genius to carry these out and with the perfect system of "interchanging," and machinery adapted for every special piece, they would be as helpless as is the so-called scientific dentist of the day. I never enter a machine-shop of the simplest character that I do not feel a reverence for men who labor and wait for recognition.

We are very far from a system that will enable us to save, with our present means, the teeth that come to us for treatment. The "American System" had no head, and it entailed upon us much that was merely experimental, to fill pages and make volumes.

I think that it was an unfortunate procedure when Dr. was first placed as a title to our names. It is like a big solitaire diamond on the hands of a man who has to labor, whether in dentistry or the trades; he cannot keep his eyes away from it. Nor can he lose sight of the fact that his hand or finger must be placed so that every one else can see it. I appreciated it as much as any one when by certain laws of the colleges a diploma was given me, which I never looked at until the officer demanded it for registration. I would prefer to have on my sign, as on my cards, my simple name.

Professor Samuel Gross said to me, "Do not complete your medical studies at the Jefferson, where you have one course to your credit, for the purpose of being with M.D.'s. Go on in your own way, and do all you can to elevate the systems of practice. Patent what you have a right to, as well as M.D.'s who copyright, learn to converse with M.D.'s intelligently on most subjects, particularly in dental practice, and I assure you that you will stand as high with us and better than a dentist who could talk medicine from the book, and with no practical experience to show in conversation."

My associates, I will not trespass longer upon your patience and feelings, but remind you that if you will but read over my article, you will find that, although not scientific, it points to the goal where we can see truth. Let us as a society, see what we can get together and place in order to show how far we are standing in the advance, and what its members have done, and place it in the Columbian Exposition, that we can measure, not swords, but the ploughshares we have made which will turn a sod that will, even when harrowed by opposition, give us a pride in our past work; and whether we are recognized as being the peer of any or forgotten, we have the consolation that

> "In doing is reward, and richest he
> Who labors not for time, but for eternity."

